# Action of Chloroform on the Blood—Sansom

**Published:** 1861-08

**Authors:** 


Foreign Items.—Dr. Elsberg gives us, in his Summary of Foreign Medical Literature, for July, (*Am. Med. Monthly*,) a resumé of Sansom's Experiments on the Action of Chloroform on the Blood.

According to the author, the effect of the mingling of liquid chloroform with the blood is an almost total solution of the red corpuscles. Under the microscope, a nearly homogeneous yellow-tinted field presents itself, with here and there only the faint outline of a red blood-corpuscle. The white corpuscles, on the other hand, appear to have been uninfluenced. To trace the most subtle effect of feebler influences, M. Sansom used in these investigations frog's blood. Placing a little undiluted on a glass slide, he exposed it for only about two seconds to the vapor proceeding from an open bottle of chloroform at the ordinary temperature of the air; having covered the blood with a slip of thin glass, he examined it under the microscope, and, as the result of his examination, he believes the progressive changes on the blood-corpuscle to be the following:

(1.) Corrugation of cell-wall; alteration of shape; coherence.
(2.) Solution of cell-wall.
(3.) Coalescence of cell contents.

To observe the effect of chloroformization of circulating blood, he exposed the web of a frog's foot on a frog-plate under the microscope, and then sprinkled a few drops of chloroform upon the damp cloth in which the frog was wrapped.

The first change noticed in the circulation was an increase of its rapidity. In about twenty seconds the capillaries had dilated to nearly twice their normal size. Then the flow became slow, the corpuscles manifested the tendency to aggregate, and, in some instances, to assume the shape which chloroform promoted in them out of the body. The cohering blood-corpuscles toiled along the capillaries in an interrupted current. More chloroform being given, the tendency to cohere became greater, the current slower. Ultimately, stasis of the blood occurred, (first in the larger vessels,) and death.

The most noticeable changes induced by chloroform on the circulating system are therefore:

(1.) Acceleration of the flow;
(2.) Dilatation of the capillaries and the blood-vessels;
(3.) Alteration in shape of blood-discs and tendency to cohere.
(4.) Stasis of the blood.

These facts have, I believe, a great influence on the theory of anæsthesia. That "narcotism is suspended oxygenation" is a doctrine receiving every day greater confirmation. I believe that the action of chloroform is directly upon the blood-corpuscle; that thereby its capability of receiving oxygen is impaired, and its faculty of stimulating the various functions of life subdued; in other words, that the phenomena of chloroformization are due, not to the agency of a circulating poison, but to the influence of an altered blood.

